# Nutrient patterns in relation to metabolic health status and serum levels of brain-derived neurotrophic factor (BDNF) and adropin in adults

**DOI:** 10.1038/s41598-024-54913-0

**Published:** 2024-02-26

**Authors:** Arghavan Balali, Shahnaz Amani Tirani, Parisa Rouhani, Farnaz Shahdadian, Zahra Hajhashemy, Sobhan Mohammadi, Elahe Mokhtari, Parvane Saneei

**Affiliations:** 1 Student Research Committee, Isfahan University of Medical Sciences, Isfahan, Iran; 2 Department of Community Nutrition, School of Nutrition and Food Science, Nutrition and Food Security Research Center, Isfahan University of Medical Sciences, Isfahan, Iran; 3 Department of Clinical Nutrition, School of Nutrition and Food Science, Nutrition and Food Security Research Center, Isfahan University of Medical Sciences, Isfahan, Iran; 4 Adelaide Medical School, Faculty of Health and Medical Sciences, University of Adelaide, Adelaide, Australia

**Keywords:** Nutrient pattern, Metabolic health, Brain-derived neurotrophic factor, Adropin, Adults, Endocrine system and metabolic diseases, Nutrition

## Abstract

The present study aimed to investigate the association of nutrient patterns (NPs) with metabolic health status and serum levels of brain-derived neurotrophic factor (BDNF) and adropin in Iranian adults. This cross-sectional survey was performed on 527 adults aged 20–60 years in Isfahan, Iran. To evaluate dietary intake, a validated 168-item semi-quantitative food frequency questionnaire (FFQ) was used. Participants were categorized as metabolically healthy (MH) and metabolically unhealthy (MU) according to their glycemic and lipid profile, insulin resistance (IR), and inflammation status. An overnight fasting blood sample was collected from each participant and serum levels of BDNF and adropin were assessed. A total of 42.50% of participants were recognized as MU. Three NPs were recognized by factor analysis that labeled as “high animal protein” (NP1), “high vegetable” (NP2), and “high carbohydrate” (NP3) patterns. Moderate adherence to NP2 was related to a lower risk of MU (OR_T2 vs. T1_ = 0.38, 95% CI: 0.18–0.76). Moreover, high adherence of NP2 (T3 vs. T1) was inversely associated with hypertriglyceridemia (OR = 0.27, 95% CI: 0.11–0.65; P-trend < 0.001) and high hs-CRP values (OR = 0.29, 95% CI: 0.09–1.00; P-trend = 0.03). No significant association was observed between adherence of NP1 and NP3 with MU in crude and adjusted models. However, negative associations were found between moderate adherence to NP3 and insulin resistance (IR) (OR = 0.23, 95% CI: 0.06–0.91) as well as high adherence to NP1 and hypertension (OR = 0.23, 95% CI: 0.09–0.61; P-trend < 0.001). NPs were not associated with serum BDNF and adropin values.

## Introduction

The concept of being metabolically healthy (MH) is defined as individuals who do not have metabolic disorders such as hypertension, some types of dyslipidemia (low levels of high-density lipoprotein (HDL) cholesterol and/or hypertriglyceridemia), and insulin resistance^1^. Although there is no etiology for metabolic health per se, it is believed that the risk of non-communicable diseases (NCDs) such as diabetes mellitus, cardiovascular diseases (CVDs), and cancers is lower in individuals with MH phenotype compared to individuals with metabolically unhealthy (MU) phenotype, even in subjects with normal weight^2^. However, MH is not a stable condition and can be converted to MU phenotype. Therefore, it is very important to find useful strategies to reduce the risk of MU phenotype and related chronic diseases.

Numerous epidemiological studies demonstrated that genetic and environmental factors, such as dietary intake, play pivotal role in the pathogenesis of MU^3^. According to the results of preceding studies, higher adherence to healthy dietary patterns prevents the conversion of MH to MU phenotype, while unhealthy eating patterns increase the risk of NCDs and inflammation^4^. Although many epidemiological studies have investigated the relationship between dietary patterns and risk of chronic diseases, the use of nutrient patterns (NPs) in this regard has some advantages. NPs can provide an appropriate tool for comparing dietary intake of different societies irrespective of what foods or food groups are consumed. NPs can also provide information about the potential mechanisms attributed to the pathogenesis of chronic diseases. These patterns are combinations of numerous nutrients that could lead to recognition of their probable synergistic effects and interactions^5^.

Some previous studies have investigated the association between NPs and metabolic complications in adults with no consistency among their reported findings. A cross-sectional study on 588 Iranian subjects (aged 18–64 years) showed a reduced risk of metabolic syndrome (MetS) in individuals with high adherence to a plant-sourced NP. However, MetS was positively associated with animal- and mixed-sourced NPs^5^. Moreover, another cross-sectional study on Iranian adults found greater odds of MetS in relation to a semi-animal NP^6^. Negative associations between risk of MetS with "saturated fatty acids, calcium, and vitamin B_2_" and "fiber, potassium, and vitamins" patterns have been found among the Japanese population^7^. However, results of the mentioned study demonstrated a positive association between MetS and "fats and fat-soluble vitamins" pattern^7^. Another investigation on Iranian women showed a positive relationship between an antioxidant pattern (containing beta-carotene, vitamin K, vitamin A, and vitamin C) and MetS^8^. Conversely, results of another cross-sectional study on 522 Iranian adults (aged 24–83 years) did not support the association between an antioxidant pattern (including omega-3 fatty acids, sodium, potassium, and lycopene) and MetS^9^. The study by Khayyatzadeh et al., involving 5764 Iranian adults, indicated a positive association between "carbohydrate, protein, starch, glucose, fructose, sucrose, and maltose" pattern and risk of MetS both in males and females. However, a pattern of "copper, selenium, vitamin A, vitamin B_2_, and vitamin B_12_" was only associated with greater odds of MetS in females^10^.

Lately, the role of biomarkers like brain-derived neurotrophic factor (BDNF) and adropin in metabolism has received a great deal of attention. Prior studies reported that these biomarkers were inversely associated with some metabolic abnormalities such as insulin resistance (IR) and dyslipidemia, through their involvement in energy hemostasis and insulin response^11,12^. In addition, there is some evidence about the effect of behavioral factors such as dietary intake on the plasma level of these biomarkers^13,14^. Based on previous findings, we hypothesized that BDNF and adropin might mediate the effect of diet on metabolic health status.

To the best of our knowledge, no previous study has evaluated the relationship between NPs and MH/MU status in adults. Thus, we aimed to address the association of NPs with metabolic health status regarding the potential role of BDNF and adropin in Iranian adults.

## Methods and materials

### Study design and population

The current population-based cross-sectional study was done in 2021 on a sample of adults aged 20–60 years residing in Isfahan, Iran. Participants were recruited from twenty schools in different educational districts of Isfahan, using a multistage cluster random-sampling method. More details regarding study design and participants have been published previously^15^. Considering an MU prevalence of 49.4% among Iranian adults^16^, confidence interval (CI) of 0.95 (type I error of 0.05), precision (d) of 4.5%, and power of 80%, minimally a sample size of 474 was required for this study. Due to high prevalence of the COVID-19 pandemic during participant recruitment, a total of 600 subjects were invited to participate in the present survey. To achieve a relatively representative sample of general adult population with diverse socioeconomic statuses, adults working in various school job categories were included in this study. Individuals were not included in the study if they: (1) were pregnant or lactating, (2) had a history of type I diabetes mellitus, stroke, CVDs, and cancer, and (3) followed a special diet. Response rate was 90.5%. Participants were excluded if they: (1) had left at least 70 items blank in their food frequency questionnaire (FFQ), (2) reported total energy intake outside the range of 800–4200 kcal/day, and (3) refused blood draw. Finally, a total of 527 subjects were included in the final analysis. An informed written consent was provided by each participant before enrollment. The study protocol was ethically approved by the local Ethics Committee of the Isfahan University of Medical Sciences, Isfahan, Iran (no. 2402203). This study was performed according to the Declaration of Helsinki^17^ and was reported based on Strengthening the Reporting of Observational Studies in Epidemiology (STROBE) guidelines^18^.

### Assessment of dietary intake

The dietary intake of participants during the previous year was evaluated using a validated 168-item semi-quantitative FFQ^19^. The validation study of this questionnaire, which was performed on 132 middle-aged adults, showed reasonable correlations between dietary intake obtained from the FFQ and twelve 24-h dietary recalls^19^. The comparison of nutrient intakes obtained from this FFQ on two occasions, 1 year apart, indicated its reliability as well. Instructions on how to complete the FFQ were given to study participants by a trained nutritionist. They were requested to report the frequency and amount of each food item they consumed in the last year. Using household measures, the portion sizes of consumed items were converted to grams per day. Afterward, to calculate total energy and nutrient intakes, all food items were transformed to the Nutritionist IV software (Version 7; First Databank, Hearst Corp, San Bruno, CA, USA). This software was based on the United States Department of Agriculture (USDA) food composition database, which was modified for Iranian food items^20^.

### Assessment of metabolic health status

Using the criteria suggested by Wildman et al.^21^, the metabolic health status of subjects was assessed. Based on this criteria, individuals with normal-weight (18.5 ≤ BMI < 25 kg/m^2^) or overweight/obesity (BMI ≥ 25 kg/m^2^) with at least two of the following risk factors were classified as metabolically unhealthy normal-weight (MUNW) and metabolically unhealthy overweight/obese (MUOW) phenotypes, respectively: (1) fasting blood glucose (FBG) levels of > 100 mg/dL; (2) HDL cholesterol levels < 40 mg/dL in males and < 50 mg/dL in females; (3) triglyceride (TG) > 150 mg/dL; (4) blood pressure (BP) > 130/85 mmHg; (5) Homeostasis Model Assessment of Insulin Resistance (HOMA-IR) > 90th percentile or > 3.99; (6) high-sensitivity C-reactive protein (hs-CRP) > 90th percentile, or > 6.14 mg/L. On the other hand, subjects with normal-weight or overweight/obesity and ≤ 1 of the aforementioned criteria were defined as metabolically healthy normal-weight (MHNW) and metabolically healthy overweight/obese (MHOW), respectively.

### Anthropometric and blood pressure measurement

Weight was measured using a body composition analyzer (Tanita MC-780MA, Tokyo, Japan) to the nearest 0.01 kg, while participants had minimal clothing and no shoes. Height assessment was done with no shoes to the nearest 0.1 cm by a non-stretched tape measure mounted on the wall. Waist circumference (WC) measurement was conducted after a normal exhalation to the nearest 0.1 cm, at the midway between the lower rib margin and the iliac crest in the standing position with no pressure on the body surface. To calculate body mass index (BMI), weight in kilogram was divided by height squared in meters (kg/m^2^). BP for each individual was assessed two times, after a resting period of 5 min in the sitting position, by using a digital sphygmomanometer (OMRON, M3, HEM-7154-E, Japan), with an accuracy of 0.5 mmHg. The mean of two measurements was recorded as the final BP.

### Assessment of biochemical indices

After 12 h of an overnight fasting, a 10-ml blood sample was collected from each participant. Blood samples were immediately centrifuged at 3500 rpm for 10 min to separate serum. These serum samples were kept at -80° C for further tests. The serum levels of FBG, TG, and HDL-cholesterol were assessed using Biosystem A15 autoanalyzer with different colorimetric methods. The commercial enzyme-linked immunosorbent assay (ELISA) kits were used for assessment of hs-CRP (turbidimetry kit, latex enhanced turbidimetric method, Delta.DP), insulin (Monobined Inc. Lake Forest, CA 92630, USA), BDNF (Zellbio, Veltlinerweg, Germany) and adropin (Zellbio, Veltlinerweg, Germany). HOMA-IR equation described by Matthews et al.^22^ was used to determine insulin resistance (IR): HOMA-IR = [FBG (mmol/L) × fasting insulin (mU/L)]/22.5.

### Assessment of other variables

Information about age, gender, education, marital status, and smoking was collected via a self-reported questionnaire. The socioeconomic status (SES) of individuals (in terms of the number of family members, home ownership, type of house, number of family cars, type of cars, job of the household head, approximate income of the family household and participant, number of laptops/computers, and number of travels in the year (within the country or abroad)) was assessed using a self-administered questionnaire. Moreover, a validated International Physical Activity Questionnaire-short form (IPAQ-SF) was used to evaluate physical activity (PA), in which individuals were classified as inactive (< 600 MET.min/week), minimally active (≥ 600 to < 3000 MET.min/week), and active (≥ 3000 MET.min/week)^23^. Furthermore, dietary habits of participants were evaluated through a pre-tested questionnaire in four domains including meal patterns, eating rate, intra-meal fluid intake, and fatty food intake^24–26^. Regarding meal patterns, individuals were asked about their eating frequency of daily main meals (breakfast, lunch, dinner) and snacks as well as the regularity of taking meals. For eating rate, participants were questioned about the chewing efficiency and time they spent on lunch and dinner. To examine intra-meal fluid intake, they were asked if they consume fluid and water with meals or immediately before and after meals. In terms of fatty food intake, participants were requested to report how many days per week they had consumed fried foods. They were also asked to report the fat content of their main meals (low fatty meals, moderate fatty meals, or high fatty meals).

### Statistical analysis

NPs were derived by inputting 35 nutrients and bioactive components (including animal protein, plant protein, vitamins B_1_, B_2_, B_3_, B_5_, B_6_, B_9_, B_12_, A, D, E, K, and C, biotin, zinc, saturated fatty acids (SFA), mono-unsaturated fatty acids (MUFA), poly-unsaturated fatty acids (PUFA), trans fatty acids (TFA), phosphorus, cholesterol, calcium, sodium, total fiber, potassium, magnesium, copper, manganese, fluoride, selenium, iron, chromium, carbohydrate, and sugar) into factor analysis with orthogonal transformation (varimax procedure). The main factors were determined based on Eigen values, Scree plot and interpretability of the factors^27^. Those factors with Eigen values > 2, as more interpretable NP, were retained in this study. To calculate factor scores for each NP, factor loading of each nutrient was first obtained, then total grams of nutrients weighted by their factor loadings were summed^27^. A factor score for each identified NP was assigned to each participant^28^. Each NP was labeled according to its high-loading nutrient groups. Afterward, subjects were classified into tertiles of NP scores. To compare quantitative (mean ± SD/SE) and categorical (percentage) variables across tertiles of NPs, one-way ANOVA and chi-square tests were used, respectively. In addition, ANCOVA test was used to assess adjusted dietary intake of participants across tertiles of NPs. Binary logistic regression was used to obtain odds ratio (OR) and 95% confidence interval (CI) for MU phenotypes across tertiles of NPs. In first model, adjustments were made for main confounding variables including age, sex and energy intake. Further adjustments for education, marital status, smoking status, SES, PA, regular eating pattern, eating rate, intra meal fluid intake, and fatty food intake were done in the second model. In the last model, additional adjustment was done for BMI^29,30^. Participants in the first tertile of NPs were considered as reference category. Tertiles of NPs were regarded as an ordinary variable to evaluate P for trend. Furthermore, stratified analyses were performed in terms of sex (female vs. male) and BMI levels (normal-weight vs. overweight/obese). BDNF values across tertiles of NPs were evaluated through linear regression analysis by controlling for age, sex, PA, high BP, high TG and high FBG. Moreover, to assess adropin levels across tertiles of NPs, linear regression analysis was applied with adjustments in terms of age, sex, energy intake, PA and BMI. All analyses were conducted using SPSS software version 26 (IBM, Chicago, IL). P-values < 0.05 were considered as statistically significant.

### Ethical approval and consent to participate

The study procedure was performed according to the declaration of Helsinki and the STROBE checklist. All participants provided informed written consent. The study protocol was approved by the local Ethics Committee of Isfahan University of Medical Sciences.

## Results

The present study was conducted on 527 Iranian adults (286 males and 241 females) with an average age (± SD) of 42.66 ± 11.19 years and a mean BMI of 26.91 ± 4.43 kg/m^2^. Three major NPs were identified among the study population by factor analysis, as shown in Supplementary Table 1. NP1 included a high intake of animal protein, cobalamin, zinc, SFA, phosphorus, riboflavin, cholesterol, MUFA, calcium, and pantothenic acid; so, it was named “high animal protein”. NP2 was characterized by high consumption of total fiber, vitamin C, potassium, TFAs, folate, vitamin A, and magnesium; therefore, it was labeled as “high vegetable”. NP3 with high intake of total fiber, thiamin, plant protein, selenium, iron, niacin, chromium, and carbohydrate was labeled “high carbohydrate”.

General characteristics of study participants across tertiles of major NPs are provided in Table 1. Participants in the third tertile of NP1 (high animal protein pattern) had a significantly higher mean of weight and FBG as well as lower mean of age in comparison to those in the first tertile (P-value < 0.05 for all cases). In addition, individuals with the highest adherence to NP1 (T3 vs. T1) were more likely to have higher SES status, educational level, and a regular eating pattern (P-value < 0.05 for all cases). In terms of NP2 (high vegetable pattern), subjects in the top tertile were more likely to have a moderate eating rate in comparison to those in the bottom tertile (P-values < 0.05). The mean age was significantly higher among adults in the highest tertile of NP2, compared to those in the lowest one (P-values < 0.05). A significant difference was observed across NP3 (high carbohydrate) tertiles regarding participants' age, sex, weight, WC, and intra-meal fluid intake (P-value < 0.05 for all cases). Individuals in the third tertile of NP3 had significantly higher systolic blood pressure and hs-CRP levels than those in the first tertile. No significant difference was seen in terms of other general features across tertiles of major NPs.

**Table 1:** General characteristics of study participants across tertiles of major NPs^1^.

	Tertiles of NP1, high animal protein			P-value^2^	Tertiles of NP2, High vegetables			P-value^2^	Tertiles of NP3, high carbohydrate			P-value^2^
	T1 (n = 175)	T2 (n = 176)	T3 (n = 176)		T1 (n = 175)	T2 (n = 176)	T3 (n = 176)		T1 (n = 175)	T2 (n = 176)	T3 (n = 176)	
Age (y)	45.48 ± 11.54	41.78 ± 11.33	40.73 ± 10.15	< 0.001	40.72 ± 10.87	42.38 ± 10.83	44.86 ± 11.52	< 0.001	42.87 ± 11.59	44.13 ± 10.33	40.98 ± 11.45	0.03
Sex (%)				0.59				0.06				< 0.001
Male	56.0	51.1	55.7		59.4	56.3	47.2		42.9	52.3	67.6	
Female	44.0	48.9	44.3		40.6	43.8	52.8		57.1	47.7	32.4	
Marital status (%)				0.83				0.96				0.31
Single	16.2	17.3	15.3		15.0	16.7	17.1		12.7	19.0	17.1	
Married	82.1	80.9	84.1		83.8	82.2	81.1		86.1	78.7	82.3	
Divorced or widow	1.7	1.7	0.6		1.2	1.1	1.7		1.2	2.3	0.6	
Education status (%)				0.04				0.98				0.56
Diploma or lower	15.5	6.9	10.9		11.4	10.9	11.0		9.8	10.3	13.1	
Higher than diploma	84.5	93.1	89.1		88.6	89.1	89.0		90.2	89.7	86.9	
Socioeconomic status (%)				< 0.001				0.12				0.67
Low	40.0	28.4	27.7		38.8	27.0	28.8		30.7	32.4	31.9	
Moderate	40.0	29.3	27.7		33.6	33.0	28.8		31.6	27.8	36.3	
High	20.0	42.2	44.5		27.6	40.0	42.3		37.7	39.8	31.9	
Weight (kg)	73.99 ± 14.57	75.37 ± 14.31	77.96 ± 14.68	0.04	76.14 ± 15.47	76.09 ± 13.39	75.10 ± 14.89	0.75	73.81 ± 13.29	74.84 ± 14.03	78.66 ± 15.94	< 0.001
BMI (kg/m^2^)	26.69 ± 4.71	26.86 ± 4.39	27.17 ± 4.21	0.59	26.68 ± 4.49	26.97 ± 3.98	27.08 ± 4.82	0.68	26.61 ± 4.22	26.79 ± 4.22	27.32 ± 4.83	0.30
Waist circumference (cm)	91.85 ± 11.45	92.33 ± 11.20	93.80 ± 11.79	0.26	92.26 ± 12.38	93.37 ± 9.99	92.36 ± 11.99	0.61	90.74 ± 11.39	92.56 ± 10.29	94.67 ± 12.42	0.01
Smoking (%)				0.90				0.80				0.24
Non-smoker	92.1	94.9	93.8		93.9	92.2	94.7		93.8	91.1	96.1	
Ex-smoker	3.9	2.5	3.1		2.4	3.9	3.3		4.4	3.8	1.3	
Current smoker	3.9	2.5	3.1		3.7	3.9	2.0		1.9	5.1	2.6	
Physical activity (MET.min/week)				0.41				0.46				0.05
Inactive	59.4	58.0	52.6		60.9	54.3	54.9		60.7	53.1	56.3	
Minimally active	32.0	33.3	41.1		33.3	35.4	37.7		31.8	42.3	32.4	
Active	8.6	8.6	6.3		5.7	10.3	7.4		7.5	4.6	11.4	
Eating pattern (%)				0.02				0.86				0.15
Regular	49.7	64.7	57.4		55.7	57.3	58.7		52.1	56.7	62.7	
Irregular	50.3	35.3	42.6		44.3	42.7	41.3		47.9	43.3	37.3	
Eating rate (%)				0.66				< 0.001				0.81
Fast	12.4	9.9	9.3		18.3	5.9	7.6		11.3	9.8	10.5	
Moderate	75.2	77.2	81.4		70.7	81.2	81.8		75.0	79.3	79.7	
Slow	12.4	12.9	9.3		11.0	12.9	10.6		13.7	11.0	9.9	
Intra meal fluid intake (%)				0.17				0.07				0.02
Moderate	70.4	64.8	60.5		59.6	64.2	71.9		71.6	66.7	57.5	
More	29.6	35.2	39.5		40.4	35.8	28.1		28.4	33.3	42.5	
Fatty food intake (%)				0.92				0.90				0.28
High	48.4	46.3	46.7		47.0	48.5	46.0		43.1	46.5	51.8	
Low	51.6	53.7	53.3		53.0	51.5	54.0		56.9	53.5	48.2	
BDNF (ng/mL)	1.43 ± 2.77	1.15 ± 0.52	1.17 ± 0.56	0.21	1.14 ± 0.48	1.49 ± 2.76	1.12 ± 0.56	0.06	1.20 ± 0.50	1.42 ± 2.76	1.13 ± 0.59	0.24
Adropin (pg/mL)	56.78 ± 44.03	56.33 ± 29.07	56.66 ± 45.67	0.99	53.84 ± 35.49	60.79 ± 41.44	55.27 ± 43.00	0.26	61.68 ± 49.84	52.57 ± 26.43	55.53 ± 40.38	0.11
Systolic blood pressure (mmHg)	122.14 ± 16.13	120.86 ± 15.71	121.97 ± 16.02	0.72	120.46 ± 16.14	122.69 ± 15.53	121.80 ± 16.14	0.42	118.99 ± 15.04	122.60 ± 15.83	123.36 ± 16.64	0.02
Diastolic blood pressure (mmHg)	83.09 ± 10.41	82.20 ± 9.22	82.51 ± 9.77	0.69	82.25 ± 10.32	83.14 ± 9.13	82.40 ± 9.95	0.66	82.11 ± 10.45	82.58 ± 8.89	83.10 ± 10.02	0.64
Fasting blood glucose (mg/dL)	91.63 ± 15.24	89.98 ± 15.90	95.29 ± 23.86	0.03	91.71 ± 17.35	91.77 ± 17.72	93.41 ± 21.28	0.63	92.41 ± 19.84	92.32 ± 18.36	92.18 ± 18.42	0.99
Triglycerides (mg/dL)	153. 61 ± 41.76	152.41 ± 40.88	153.70 ± 39.97	0.95	156.82 ± 43.89	153.19 ± 41.75	149.73 ± 36.33	0.27	149.31 ± 38.45	153.76 ± 40.63	156.64 ± 43.07	0.24
HDL-c (mg/dL)	55.12 ± 9.99	55.79 ± 9.80	55.26 ± 10.21	0.80	54.64 ± 10.90	56.22 ± 9.30	55.30 ± 9.69	0.33	55.88 ± 10.22	56.34 ± 9.72	53.95 ± 9.90	0.06
hs-CRP (> 90th percentile) (mg/L)	2.98 ± 2.25	3.40 ± 3.32	3.17 ± 3.08	0.41	3.20 ± 2.90	3.16 ± 2.98	3.19 ± 2.90	0.99	3.57 ± 3.23	2.73 ± 2.52	3.26 ± 2.92	0.02
HOMA-IR index (> 90th percentile)	1.69 ± 2.78	1.68 ± 2.63	1.52 ± 1.83	0.75	1.58 ± 2.43	1.61 ± 2.49	1.71 ± 2.42	0.89	1.90 ± 3.02	1.36 ± 1.72	1.64 ± 2.40	0.11

*y* year, *kg* kilogram, *mg* milligram, *m* meter, *cm* centimeter, *BMI* body mass index, *n* number, *mmHg* millimeter Hg, *ng* nanogram, *pg* pictogram, *mL* milliliter, *dL* deciliter, *NP* nutrient pattern, *T* tertile.

^1^Values are Mean ± SD; unless indicated.

^2^Obtained from ANOVA and χ^2^ test for quantitative and categorical variables, respectively.

Daily dietary intake of study participants across tertiles of major NPs is shown in Table 2. Adults in the top tertile of NP1 (high animal protein) had a higher intake of energy, protein, total fat, saturated fatty acids (SFAs), calcium, dairy, red and processed meat, and white meat compared with those in the bottom tertile (P-value < 0.05 for all cases). However, intake of carbohydrates, vitamin C, iron, total fiber, fruits, whole grains, and refined grains was lower in subjects with the highest adherence of NP1 in comparison to those with the lowest adherence (T3 vs. T1) (P-value < 0.05 for all cases). In the case of NP2 (high vegetable), participants in the third tertile had significantly higher intake of energy, carbohydrate, vitamin C, vitamin B_6_, vitamin B_9_, vitamin E, total fiber, vegetables, fruits, legumes, and nuts than those in the first tertile (P-value < 0.05 for all cases). In comparison with adults in the first tertile of NP2, those in the third tertile had lower consumption of protein, total fat, SFAs, whole grains, refined grains, and red/processed meats (P-value < 0.05 for all cases). Regarding NP3 (high carbohydrate), subjects with the highest score had higher intake of energy, carbohydrates, iron, whole grains, and refined grains than those with the lowest score (P-value < 0.05 for all cases). However, lower intake of protein, total fat, SFAs, vitamin C, vitamin B_9_, vitamin E, calcium, total fiber, sodium, vegetables, fruits, dairy, and white meat was observed for participants with the highest adherence of NP3 than those with the lowest adherence (P-values < 0.05 for all cases).

**Table 2:** Daily dietary intake of study participants across tertiles of major NPs^1^.

	Tertiles of NP1, high animal protein			P-value^2^	Tertiles of NP2, high vegetable			P-value^2^	Tertiles of NP3, high carbohydrate			P-value^2^
	T1 (n = 175)	T2 (n = 176)	T3 (n = 176)		T1 (n = 175)	T2 (n = 176)	T3 (n = 176)		T1 (n = 175)	T2 (n = 176)	T3 (n = 176)	
Energy, kcal	1897.07 ± 44.53	2191.12 ± 43.99	2740.58 ± 44.17	< 0.001	1922.93 ± 45.09	2178.01 ± 44.63	2727.97 ± 45.14	< 0.001	1795.06 ± 41.79	2238.41 ± 41.42	2794.71 ± 42.06	< 0.001
Protein, % of E	12.42 ± 0.19	14.23 ± 0.18	16.08 ± 0.19	< 0.001	15.06 ± 0.21	14.04 ± 0.21	13.65 ± 0.21	< 0.001	15.05 ± 0.21	14.20 ± 0.21	13.50 ± 0.21	< 0.001
Carbohydrate, % of E	67.69 ± 0.46	60.85 ± 0.46	54.22 ± 0.46	< 0.001	58.26 ± 0.60	60.58 ± 0.59	63.87 ± 0.60	< 0.001	59.12 ± 0.61	60.95 ± 0.61	62.64 ± 0.62	< 0.001
Fat, % of E	21.93 ± 0.42	26.90 ± 0.41	31.55 ± 0.41	< 0.001	27.77 ± 0.50	27.32 ± 0.50	25.33 ± 0.50	< 0.001	28.05 ± 0.50	26.74 ± 0.50	25.63 ± 0.51	< 0.001
Saturated fatty acid, gr	15.99 ± 0.49	21.05 ± 0.46	29.84 ± 0.50	< 0.001	25.04 ± 0.60	22.60 ± 0.57	19.30 ± 0.62	< 0.001	26.23 ± 0.62	22.72 ± 0.55	17.98 ± 0.63	< 0.001
Vitamin C, mg	216.05 ± 8.15	204.39 ± 7.57	175.01 ± 8.33	< 0.001	115.22 ± 6.24	181.95 ± 5.86	297.70 ± 6.44	< 0.001	251.67 ± 8.18	189.87 ± 7.24	154.10 ± 8.35	< 0.001
Pyridoxine, mg	1.75 ± 0.04	1.83 ± 0.04	1.82 ± 0.04	0.30	1.49 ± 0.04	1.79 ± 0.03	2.13 ± 0.04	< 0.001	1.97 ± 0.04	1.78 ± 0.04	1.66 ± 0.04	0.05
Vitamin E, mg	7.22 ± 0.25	6.53 ± 0.23	6.89 ± 0.26	0.12	5.69 ± 0.24	6.83 ± 0.23	8.11 ± 0.25	< 0.001	7.43 ± 0.26	6.76 ± 0.23	6.44 ± 0.27	< 0.001
Folate, mcg	336.38 ± 9.12	341.94 ± 8.48	347.24 ± 9.33	0.74	263.84 ± 7.62	328.60 ± 7.16	432.70 ± 7.87	< 0.001	369.71 ± 9.46	337.31 ± 8.38	318.73 ± 9.65	< 0.001
Iron, mg	19.75 ± 0.31	17.81 ± 0.29	16.03 ± 0.32	< 0.001	18.41 ± 0.32	17.65 ± 0.31	17.51 ± 0.34	0.13	15.19 ± 0.31	17.50 ± 0.27	20.87 ± 0.31	< 0.001
Calcium, mg	717.24 ± 27.27	873.04 ± 25.33	1180.64 ± 27.88	< 0.001	903.21 ± 29.87	889.11 ± 28.09	979.66 ± 30.86	0.09	1176.37 ± 28.22	953.27 ± 24.99	643.89 ± 28.80	< 0.001
Total fiber, gr	23.36 ± 0.50	21.72 ± 0.47	18.43 ± 0.51	< 0.001	15.42 ± 0.37	20.12 ± 0.35	27.93 ± 0.38	< 0.001	22.97 ± 0.54	21.14 ± 0.48	19.40 ± 0.55	< 0.001
Sodium, mg	3363.91 ± 205.53	3830.06 ± 190.91	4129.75 ± 210.18	0.05	3575.60 ± 203.86	3754.55 ± 191.69	3994.77 ± 210.62	0.41	4185.17 ± 214.70	3952.11 ± 190.14	3191.10 ± 219.11	0.01
Food groups												
Vegetables, g	348.40 ± 18.22	341.70 ± 16.92	334.41 ± 18.63	0.88	211.54 ± 15.61	294.54 ± 14.68	517.65 ± 16.13	< 0.001	407.73 ± 18.65	356.58 ± 16.51	260.53 ± 19.03	< 0.001
Fruits, g	653.93 ± 25.27	568.22 ± 23.47	446.44 ± 25.84	< 0.001	329.63 ± 21.47	517.51 ± 20.19	819.60 ± 22.19	< 0.001	724.09 ± 25.65	546.91 ± 22.71	398.00 ± 26.17	< 0.001
Dairy, g	169.09 ± 19.36	278.42 ± 17.99	501.71 ± 19.80	< 0.001	322.15 ± 21.36	305.79 ± 20.08	322.16 ± 22.06	0.80	472.16 ± 20.63	343.68 ± 18.27	135.11 ± 21.05	< 0.001
Whole grains, g	142.84 ± 6.31	107.96 ± 5.86	86.91 ± 6.46	< 0.001	134.42 ± 6.32	112.04 ± 5.94	91.21 ± 6.53	< 0.001	70.62 ± 6.37	106.62 ± 6.64	160.05	< 0.001
Refined grain, g	326.61 ± 12.74	279.72 ± 11.83	214.88 ± 13.03	< 0.001	316.06 ± 12.69	282.26 ± 11.94	222.83 ± 13.11	< 0.001	158.84 ± 11.89	253.77 ± 10.53	407.64 ± 12.13	< 0.001
Legumes, g	37.06 ± 2.98	42.66 ± 2.77	38.02 ± 3.05	0.31	30.02 ± 2.91	42.54 ± 2.74	45.15 ± 3.01	< 0.001	36.17 ± 3.12	38.35 ± 2.77	43.21 ± 3.19	0.35
Nuts, g	10.17 ± 1.02	11.63 ± 0.95	13.70 ± 1.05	0.08	9.74 ± 1.01	13.02 ± 0.95	12.74 ± 1.04	0.04	12.36 ± 1.08	12.08 ± 0.95	11.07 ± 1.10	0.72
Red and processed Meat, g	42.40 ± 3.32	62.73 ± 3.08	98.17 ± 3.39	< 0.001	83.61 ± 3.53	66.80 ± 3.32	53.12 ± 3.65	< 0.001	71.72 ± 3.86	66.41 ± 3.42	65.34 ± 3.94	0.50
White meat, g	24.48 ± 2.57	39.32 ± 2.39	51.84 ± 2.63	< 0.001	42.30 ± 2.65	39.03 ± 2.49	34.41 ± 2.73	0.15	45.76 ± 2.79	39.39 ± 2.47	30.62 ± 2.84	< 0.001

*g* gram, *mg* milligram, *mcg* microgram, *NP* nutrient pattern, *T* tertile.

^1^Values are Mean ± SE. Energy intake and macronutrients were adjusted for age and sex; all other values were adjusted for age, sex and energy intake.

^2^Obtained from ANCOVA.

Out of 527 study participants, 224 adults (147 males and 77 females) were characterized as MU. Multivariate adjusted odds ratio (OR) and 95% confidence interval (CI) for MU profile across tertiles of major NPs are presented in Table 3. Regarding NP2 (high vegetable), adults in the second tertile had significantly lower odds of MU profile than those in the first tertile. Such that, in the fully adjusted model, participants in the top tertile vs. the bottom tertile had 62% lower odds of MU profile (OR = 0.38, 95% CI: 0.18–0.76). In terms of NP1 (high animal protein) and NP3 (high carbohydrate), no significant association was observed with MU phenotype, either in crude or adjusted models.

**Table 3:** Multivariate adjusted odds ratio (OR) and 95% confidence interval (CI) for MU profile across tertiles of major NPs^1^.

	T1 (n = 175)	T2 (n = 176)	T3 (n = 176)	P-trend^2^
Tertiles of NP1 (High animal protein)				
Cases (n)	74	72	78	
Crude	1 (Ref)	0.95 (0.62, 1.45)	1.09 (0.71, 1.66)	0.70
Model 1^3^	1 (Ref)	1.15 (0.72, 1.83)	1.29 (0.76, 2.18)	0.35
Model 2^4^	1 (Ref)	0.91 (0.42, 1.95)	0.83 (0.35, 1.98)	0.68
Model 3^5^	1 (Ref)	0.98 (0.45, 2.15)	0.90 (0.37, 2.18)	0.81
Tertiles of NP2 (High vegetable)				
Cases (n)	75	67	82	
Crude	1 (Ref)	0.82 (0.54, 1.26)	1.16 (0.76, 1.77)	0.48
Model 1^3^	1 (Ref)	0.69 (0.44, 1.10)	0.84 (0.50, 1.41)	0.45
Model 2^4^	1 (Ref)	0.39 (0.20, 0.79)	0.65 (0.28, 1.48)	0.16
Model 3^5^	1 (Ref)	0.38 (0.18, 0.76)	0.60 (0.26, 1.40)	0.12
Tertiles of NP3 (High carbohydrate)				
Cases (n)	73	78	73	
Crude	1 (Ref)	1.11 (0.73, 1.70)	0.99 (0.65, 1.51)	0.96
Model 1^3^	1 (Ref)	0.82 (0.51, 1.33)	0.62 (0.35, 1.10)	0.11
Model 2^4^	1 (Ref)	0.71 (0.33, 1.51)	1.08 (0.45, 2.59)	0.86
Model 3^5^	1 (Ref)	0.71 (0.32, 1.53)	1.08 (0.44, 2.63)	0.85

*NP* nutrient pattern, *MU* metabolically unhealthy, *T* tertile, *n* number.

^1^All values are odds ratios and 95% confidence intervals.

^2^Obtained by the use of tertiles of major nutrient patterns as an ordinal variable in the model.

^3^Model 1: Adjusted for age, sex, and energy intake.

^4^Model 2: Additionally adjusted for marital status, education, smoking status, socioeconomic status, physical activity, regular eating pattern, eating rate, intra meal fluid intake, and fatty food intake.

^5^Model 3: More adjustments for body mass index (BMI).

Multivariate adjusted odds ratio (OR) and 95% confidence interval (CI) for individual components of MU across tertiles of NPs are depicted in Table 4. After adjustment for potential confounders, participants in top tertile of NP1 (high animal protein) had 77% lower odds of hypertension (OR = 0.23, 95% CI: 0.09–0.61; P-trend = 0.01) than those in first tertile. No significant difference was seen in terms of other components of MU across tertiles of NP1. Adults with the highest adherence to NP2 (high vegetable) had respectively 73% and 71% lower odds of hypertriglyceridemia (OR = 0.27, 95% CI: 0.11–0.65; P-trend = 0.01) and high hs-CRP values (OR = 0.29, 95% CI: 0.09–1.00; P-trend = 0.03), compared to those with the lowest adherence, in fully-adjusted model. In addition, subjects with moderate adherence to NP2 (T2) had 60% lower odds of low HDL-cholesterolemia (OR = 0.40, 95% CI: 0.18–0.86) than those with low adherence (T1), in crude model. After taking potential confounders into account, this association became non-significant (OR = 0.32, 95% CI: 0.10–1.02). In case of NP3, adults with moderate adherence to NP3 (high carbohydrate) had 68% higher odds of hypertension (OR = 1.68, 95% CI: 1.10–2.56), compared to those with low adherence, in crude model. However, after considering confounders, this association disappeared. Moreover, after controlling for potential confounders, a 77% lower odds of IR (OR = 0.23, 95% CI: 0.06–0.91) was observed for participants in the second category of NP3 than those in the first category.

**Table 4:** Multivariate adjusted odds ratio (OR) and 95% confidence interval (CI) for individual components of MU across tertiles of major NPs^1^.

	Tertiles of NP1, high animal protein			P-trend^3^	Tertiles of NP2, high vegetable			P-trend^3^	Tertiles of NP3, high carbohydrate			P-trend^3^
	T1 (n = 175)	T2 (n = 176)	T3 (n = 176)		T1 (n = 175)	T2 (n = 176)	T3 (n = 176)		T1 (n = 175)	T2 (n = 176)	T3 (n = 176)	
High FBG levels												
Cases	35	27	42		34	34	36		35	38	31	
Crude	1 (Ref)	0.73 (0.42, 1.26)	1.25 (0.76, 2.08)	0.36	1 (Ref)	0.99 (0.59, 1.69)	1.07 (0.63, 1.80)	0.81	1 (Ref)	1.10 (0.66, 1.85)	0.86 (0.50, 1.46)	0.57
Fully-adjusted model^2^	1 (Ref)	0.81 (0.31, 2.10)	1.32 (0.46, 3.81)	0.55	1 (Ref)	0.71 (0.31, 1.63)	0.86 (0.33, 2.27)	0.69	1 (Ref)	1.38 (0.56, 3.42)	1.51 (0.51, 4.46)	0.46
Low HDL-c levels												
Cases	25	18	18		23	10	28		20	18	23	
Crude	1 (Ref)	0.68 (0.36, 1.30)	0.68 (0.36, 1.30)	0.24	1 (Ref)	0.40 (0.18, 0.86)	1.25 (0.69, 2.27)	0.42	1 (Ref)	0.88 (0.45, 1.73)	1.17 (0.62, 2.21)	0.63
Fully-adjusted model^2^	1 (Ref)	1.04 (0.33, 3.30)	0.50 (0.14, 1.83)	0.27	1 (Ref)	0.32 (0.10, 1.02)	0.84 (0.27, 2.67)	0.62	1 (Ref)	0.55 (0.14, 2.13)	2.12 (0.60, 7.49)	0.24
High TG levels												
Cases	60	63	70		70	63	60		56	70	67	
Crude	1 (Ref)	1.07 (0.69, 1.66)	1.27 (0.82, 1.95)	0.29	1 (Ref)	0.84 (0.54, 1.29)	0.78 (0.50, 1.20)	0.25	1 (Ref)	1.40 (0.91, 2.18)	1.31 (0.84, 2.03)	0.24
Fully-adjusted model^2^	1 (Ref)	1.11 (0.49, 2.48)	1.91 (0.78, 4.67)	0.14	1 (Ref)	0.51 (0.25, 1.04)	0.27 (0.11, 0.65)	0.01	1 (Ref)	1.30 (0.59, 2.85)	1.42 (0.57, 3.54)	0.45
Hypertension												
Cases	92	80	77		78	86	85		72	95	82	
Crude	1 (Ref)	0.75 (0.49, 1.14)	0.70 (0.46, 1.07)	0.10	1 (Ref)	1.19 (0.78, 1.81)	1.16 (0.76, 1.77)	0.49	1 (Ref)	1.68 (1.10, 2.56)	1.25 (0.82, 1.90)	0.31
Fully-adjusted model^2^	1 (Ref)	0.34 (0.15, 0.78)	0.23 (0.09, 0.61)	0.01	1 (Ref)	0.51 (0.24, 1.08)	1.28 (0.54, 3.03)	0.81	1 (Ref)	1.90 (0.85, 4.22)	1.58 (0.64, 3.92)	0.32
High HOMA-IR												
Cases	16	18	18		16	17	19		19	15	18	
Crude	1 (Ref)	1.13 (0.56, 2.30)	1.13 (0.56, 2.30)	0.73	1 (Ref)	1.06 (0.52, 2.18)	1.20 (0.60, 2.42)	0.60	1 (Ref)	0.77 (0.38, 1.56)	0.94 (0.47, 1.85)	0.84
Fully-adjusted model^2^	1 (Ref)	1.17 (0.31, 3.67)	0.91 (0.24, 3.52)	0.87	1 (Ref)	1.43 (0.50, 4.13)	2.61 (0.74, 9.22)	0.14	1 (Ref)	0.23 (0.06, 0.91)	0.96 (0.26, 3.59)	0.88
High CRP levels												
Cases	12	23	17		21	14	17		22	12	18	
Crude	1 (Ref)	2.04 (0.98, 4.25)	1.45 (0.67, 3.14)	0.38	1 (Ref)	0.63 (0.31, 1.29)	0.78 (0.40, 1.54)	0.46	1 (Ref)	0.51 (0.24, 1.06)	0.79 (0.41, 1.54)	0.46
Fully-adjusted model^2^	1 (Ref)	2.53 (0.72, 8.83)	2.90 (0.71, 11.87)	0.16	1 (Ref)	0.39 (0.14, 1.12)	0.29 (0.09, 1.00)	0.03	1 (Ref)	0.37 (0.11, 1.22)	0.87 (0.25, 3.07)	0.72

*NP* nutrient pattern, *MU* metabolically unhealthy, *T* tertile, *n* number.

^1^All values are odds ratios and 95% confidence intervals.

^2^Fully adjusted model: Adjusted for age, sex, energy intake, marital status, education, smoking status, socioeconomic status, physical activity, regular eating pattern, eating rate, intra meal fluid intake, fatty food intake, and BMI.

^3^Obtained by the use of tertiles of major nutrient patterns as an ordinal variable in the model.

Multivariate adjusted ORs and 95%CI for MU profile across tertiles of major NPs, stratified by sex and BMI categories are provided in Table 5. In crude or adjusted models, no significant association was seen between NP1 (high animal protein) and MU phenotype in both males and females. In the case of NP2 (high vegetable), after adjustment for confounding variables, males with moderate adherence, compared to those with lower adherence (T2 vs. T1), had 77% lower odds of MU profile (OR = 0.23, 95% CI: 0.08–0.64). No significant relation was observed between NP2 and MU in females. Regarding NP3 (high carbohydrate), high adherence to this pattern was associated with lower odds of MU profile in males (OR = 0.50, 95% CI: 0.27–0.89; P-trend = 0.02), in crude model; however, after taking potential confounders into account, this association disappeared (OR = 0.42, 95% CI: 0.10–1.72; P-trend = 0.31). Although no significant association was seen between NP3 and MU profile for females in crude model, after adjustment for confounders, the highest adherence to NP3 compared to the lowest adherence was associated with 5.43 times higher likelihood of MU phenotype (OR = 5.43, 95% CI: 1.13–26.13; P-trend = 0.04).

**Table 5:** Multivariate adjusted odds ratio (OR) and 95% confidence interval (CI) for MU profile across tertiles of major NPs, stratified by sex and BMI categories^1^.

	Tertiles of NP1, high animal proteins			P-trend^5^	Tertiles of NP2, high vegetable			P-trend^5^	Tertiles of NP3, high carbohydrate			P-trend^5^
	T1	T2	T3		T1	T2	T3		T1	T2	T3	
Males												
Participants/cases	98/50	90/48	98/49		104/53	99/46	83/48		75/47	92/46	119/54	
Crude	1 (Ref)	1.10 (0.62, 1.95)	0.96 (0.55, 1.68)	0.89	1 (Ref)	0.84 (0.48, 1.45)	1.32 (0.74, 2.36)	0.40	1 (Ref)	0.60 (0.32, 1.11)	0.50 (0.27, 0.89)	0.02
Model 1^2^	1 (Ref)	1.37 (0.73, 2.57)	1.32 (0.66, 2.64)	0.42	1 (Ref)	0.66 (0.36, 1.19)	0.82 (0.40, 1.67)	0.48	1 (Ref)	0.50 (0.25, 1.01)	0.39 (0.18, 0.88)	0.03
Model 2^3^	1 (Ref)	1.36 (0.45, 4.15)	1.31 (0.37, 4.60)	0.72	1 (Ref)	0.29 (0.11, 0.75)	0.62 (0.19, 1.98)	0.21	1 (Ref)	0.32 (0.10, 1.01)	0.44 (0.11, 1.73)	0.32
Model 3^4^	1 (Ref)	1.50 (0.48, 4.73)	1.33 (0.37, 4.76)	0.74	1 (Ref)	0.23 (0.08, 0.64)	0.58 (0.17, 1.90)	0.16	1 (Ref)	0.30 (0.09, 1.01)	0.42 (0.10, 1.72)	0.31
Females												
Participants/cases	77/24	86/24	78/29		71/22	77/21	93/34		100/26	84/32	57/19	
Crude	1 (Ref)	0.86 (0.44, 1.68)	1.31 (0.67, 2.54)	0.42	1 (Ref)	0.84 (0.41, 1.70)	1.28 (0.67, 2.48)	0.40	1 (Ref)	1.75 (0.94, 3.28)	1.42 (0.70, 2.89)	0.24
Model 1^2^	1 (Ref)	0.95 (0.46, 1.93)	1.25 (0.55, 2.83)	0.62	1 (Ref)	0.75 (0.35, 1.58)	0.84 (0.38, 1.86)	0.69	1 (Ref)	1.28 (0.65, 2.52)	0.97 (0.40, 2.33)	0.95
Model 2^3^	1 (Ref)	0.50 (0.13, 1.91)	0.36 (0.08, 1.71)	0.20	1 (Ref)	0.38 (0.10, 1.36)	0.36 (0.08, 1.58)	0.15	1 (Ref)	2.05 (0.55, 7.60)	5.49 (1.17, 25.69)	0.03
Model 3^4^	1 (Ref)	0.58 (0.15, 2.30)	0.47 (0.09, 2.39)	0.37	1 (Ref)	0.38 (0.10, 1.42)	0.33 (0.07, 1.48)	0.13	1 (Ref)	2.17 (0.56, 8.41)	5.43 (1.13, 26.13)	0.04
Normal-weight participants												
Participants/cases	60/18	59/17	51/11		63/17	57/14	50/15		60/11	61/24	49/11	
Crude	1 (Ref)	0.94 (0.43, 2.08)	0.64 (0.27, 1.53)	0.33	1 (Ref)	0.88 (0.39, 2.00)	1.16 (0.51, 2.64)	0.75	1 (Ref)	2.89 (1.26, 6.64)	1.29 (0.51, 3.29)	0.52
Model 1^2^	1 (Ref)	1.39 (0.58, 3.33)	1.03 (0.35, 3.04)	0.89	1 (Ref)	1.02 (0.41, 2.52)	1.33 (0.47, 3.74)	0.60	1 (Ref)	2.65 (1.09, 6.45)	1.24 (0.39, 3.89)	0.58
Model 2^3^	1 (Ref)	0.79 (0.14, 4.65)	0.95 (0.14, 6.57)	0.99	1 (Ref)	0.19 (0.04, 1.06)	0.20 (0.03, 1.58)	0.10	1 (Ref)	5.01 (0.81, 30.94)	3.09 (0.30, 32.05)	0.33
Overweight/obese participants												
Participants/cases	115/56	117/55	125/67		112/58	119/53	126/67		115/62	115/54	127/62	
Crude	1 (Ref)	0.94 (0.56, 1.57)	1.22 (0.73, 2.02)	0.44	1 (Ref)	0.75 (0.45, 1.26)	1.06 (0.64, 1.76)	0.79	1 (Ref)	0.76 (0.45, 1.27)	0.82 (0.49, 1.35)	0.44
Model 1^2^	1 (Ref)	1.00 (0.57, 1.76)	1.30 (0.69, 2.44)	0.42	1 (Ref)	0.59 (0.34, 1.03)	0.75 (0.40, 1.40)	0.31	1 (Ref)	0.51 (0.28, 0.93)	0.45 (0.22, 0.91)	0.03
Model 2^3^	1 (Ref)	0.78 (0.29, 2.05)	0.56 (0.18, 1.73)	0.31	1 (Ref)	0.23 (0.09, 0.60)	0.64 (0.22, 1.90)	0.18	1 (Ref)	0.30 (0.11, 0.85)	0.98 (0.32, 3.00)	0.92

*NP* nutrient pattern, *MU* metabolically unhealthy, *T* tertile.

^1^All values are odds ratios and 95% confidence intervals.

^2^Model 1: Adjusted for age, sex, and energy intake. In stratified analysis by sex, adjusted for age and energy intake.

^3^Model 2: Additionally adjusted for marital status, education, smoking status, socioeconomic status, physical activity, regular eating pattern, eating rate, intra meal fluid intake, and fatty food intake.

^4^Model 3: Additionally adjusted for BMI.

^5^Obtained by the use of tertiles of major nutrient patterns as an ordinal variable in the model.

Stratified analysis by BMI categories (Table 5) revealed no relation between NP1 and MU profile, either in individuals with normal-weight or overweight/obesity. In the case of NP2, subjects with overweight/obesity in the second category of NP2 had a 77% lower odds of MU profile (OR = 0.23, 95% CI: 0.09–0.60), in comparison to those in the reference category. In terms of NP3, after adjustment for potential confounders, subjects with overweight/obesity with moderate adherence had 70% lower odds of MU profile (OR = 0.30, 95% CI: 0.11–0.85) than those with low adherence. However, no significant association was found between NP3 and MU phenotype among adults with normal-weight, in maximally-adjusted model.

Linear associations between each tertile increase in major NPs with serum BDNF and adropin levels are depicted in Table 6. Either in crude or adjusted models, each tertile increase in NPs was not significantly associated with serum BDNF levels (P-values > 0.05). Similar findings were also observed in terms of circulating adropin concentrations (P-values > 0.05). Although NP2 (high vegetable) and NP3 (high carbohydrate) were respectively associated with higher and lower values of adropin, these relations were not statistically significant.

**Table 6:** Linear association between each tertile increase in major NPs with serum BDNF and adropin levels^1^.

	Per 1 tertile increase in NP1 adherence, high animal protein				Per 1 tertile increase in NP2 adherence, high vegetable				Per 1 tertile increase in NP3 adherence, high carbohydrate			
	B	95% CI	P-value	R^2^	B	95% CI	P-value	R^2^	B	95% CI	P-value	R^2^
BDNF (n = 527)												
Crude	− 1.30	1.14, 1.89	0.14	< 0.001	− 0.01	− 0.18, 0.17	0.92	< 0.001	− 0.03	− 0.20, 0.14	0.73	< 0.001
Model 1^2^	− 0.11	− 0.29, − 0.07	0.22	0.01	− 0.03	− 0.21, 0.15	0.73	< 0.001	− 0.02	− 0.20, 0.16	0.85	< 0.001
Model 2^3^	− 0.11	− 0.28, 0.07	0.25	0.01	− 0.04	− 0.22, 0.14	0.68	0.01	− 0.03	− 0.21, 0.15	0.77	0.01
Adropin (n = 497)												
Crude	− 0.05	− 4.41, 4.31	0.98	< 0.001	0.71	− 3.60, 5.02	0.75	< 0.001	− 3.09	− 7.42, 1.25	0.16	< 0.001
Model 1^4^	1.51	− 3.55, 6.58	0.56	0.01	2.55	− 2.46, 7.55	0.32	0.01	− 1.32	− 6.83, 4.19	0.64	0.01
Model 2^5^	1.65	− 3.45, 6.74	0.53	0.01	2.57	− 2.47, 7.60	0.32	0.01	− 1.39	− 6.94, 4.17	0.62	0.01

*NP* nutrient pattern, *n* number, *BDNF* brain-derived neurotrophic factor.

^1^All values are regression coefficients and 95% confidence intervals. Tertiles of major NPs were considered as an ordinal variable.

^2^Adjusted for age and sex.

^3^Adjusted for age, sex, physical activity, fasting blood glucose, triglyceride, and blood pressure.

^4^Adjusted for age and sex, and total energy intake.

^5^Adjusted for age and sex, total energy intake, physical activity, and BMI.

## Discussion

In the present study, three NPs were recognized and labeled as “high animal protein” (NP1), “high vegetable” (NP2), and “high carbohydrate” (NP3) patterns. The present survey indicated that moderate adherence to the “high vegetable” pattern (T2 vs. T1) was associated with lower odds of MU profile particularly in males and subjects with overweight/obesity. A lower likelihood of having hypertriglyceridemia and chronic inflammation was also observed in individuals with a high adherence to “high vegetable” patterns (T3 vs. T1). No significant association has been found between NP1 and NP3 with MU risk. However, inverse associations were found between high adherence to the “high animal protein” pattern (T3 vs. T1) and hypertension, and also between moderate adherence to the “high carbohydrate” pattern (T2 vs. T1) and IR.

The findings of the current study revealed that the risk of MU profile can be ameliorated by moderate intake of nutrients found in the "high vegetable" pattern in Iranian adults. Therefore, moderate consumption of foods containing nutrients like dietary fiber, vitamin C, potassium, folate, vitamin A, and magnesium can be recommended to the general population to decrease the risk of MU.

The current survey found that moderate adherence to the “high vegetable” pattern was associated with a lower risk of MU profile, mainly in males and subjects with obesity/overweight. Evidence from different populations indicates that higher intakes of dietary fiber^31^, potassium^32^, magnesium^33^, vitamin C^34^, folate^35^, and vitamin A^36^ are negatively associated with odds of MetS. However, TFAs intake adversely affects metabolic health status^37^. The inverse association between moderate intake of nutrients loaded in the "high vegetable" pattern and MU indicates that interactions between protective nutrients and TFAs could decrease the risk of MU. However, results of the present study failed to show any significant association between high adherence to “high vegetable pattern” (T3 vs. T1) and MU risk. It is postulated that protective nutrients possibly could not alleviate the unfavorable effects of MU-induced nutrients in individuals with high adherence to the mentioned pattern. Furthermore, use of pesticides and presence of heavy metals in soil where plant foods, as the main sources of these nutrients, are cultivated could be factors that had negative effects on metabolic status of individuals with the highest adherence to "high vegetable" pattern in Iranian population^38^. The beneficial effects of the "high vegetable" pattern on metabolic health status could be justified by some mechanisms. For example, some of the nutrients in this pattern such as magnesium^39^, vitamin C^40^, fiber^41,42^, and folate^42^ could suppress inflammation, which has been associated with metabolic health status^43,44^. Increased serum levels of pro-inflammatory cytokines such as tumor necrosis factor-alpha (TNF-alpha) and interleukin-6 (IL-6) have been documented in patients with metabolic disorders. Pro-inflammatory cytokines could play roles in insulin resistance through different mechanisms such as inducing inflammatory pathways or insulin signaling pathway modification^45,46^. Insulin resistance has also been known to play a key role in the pathogenesis of cardiometabolic disorders^45,46^. The favorable role of dietary magnesium and fiber on insulin resistance has also been reported previously^47–51^. Further studies are needed to determine the exact mechanisms underlying this association.

The unfavorable role of several nutrients of "high animal protein" like animal protein^52^, SFAs^53^, and cholesterol^54^ on metabolic health status has been demonstrated in previous research. Whereas, a negative link has been detected between the risk of MetS with dietary intake of other nutrients of this pattern such as calcium^55^ and MUFA^56^. We did not discover any significant relationship between high adherence to "high animal protein" pattern and MU odds in the present study. However, it was found that higher adherence to the pattern was linked to a reduced risk of hypertension. The protective effect of the “high animal protein” pattern on BP might be addressed by the antihypertensive effect of some amino acids in animal proteins (such as arginine, tyrosine, and tryptophan^57,58^), as well as calcium^59^, and vitamin B_12_^60^. The favorable and unfavorable effects of nutrients loaded in the "high carbohydrate" pattern on metabolic health status have also been investigated before^61–66^. In the current study, no significant relationship was found between the "high carbohydrate" pattern and MU phenotype, although high adherence to this pattern was associated with an increased risk of MU among females. Some factors such as nutrients deficiency (for example, iron and calcium deficiencies) and higher insulin sensitivity in skeletal muscles in females might explain the observed relationship^67,68^. However, the controversial effects of various nutrients on metabolic health status besides their interactions make it difficult to interpret these findings. Further large-scale studies in various populations are warranted to explore the association between various NPs and metabolic health status precisely.

The present study was among the first studies that investigated the relationship between NPs and metabolic health status in a relatively representative sample of Iranian adults. Moreover, the assessment of outcome of interest was based on objective assessments rather than self-reported data. Additionally, the definition used for metabolic health status was considered IR and chronic inflammation. Also, several potential confounders (such as socio-demographic variables, smoking, physical activity, total energy intake, BMI, and dietary habit domains) were considered in statistical analyses to find independent relations. However, some limitations should be considered when interpreting these results. First, according to cross-sectional design of the present study, causal inference between NPs and metabolic health status cannot be discerned. Further prospective studies are warranted to affirm causality. In addition, the effect of the COVID-19 pandemic on dietary intake, physical activity, and metabolic health status cannot be ignored. In the current study, no significant link was found between serum levels of BDNF and adropin with NPs, which can be explained by our relatively small sample size, due to limited financial resources. Also, like other epidemiological studies, misclassification of the study participants (due to the use of a FFQ) was unavoidable; recall bias might also affect findings despite using a validated FFQ. The last but not the least, the effect of residual and unknown confounders on findings should be considered.

## Conclusion

The present investigation highlighted that moderate adherence to the “high vegetable” pattern was inversely associated with a lower likelihood of MU in Iranian adults. The association was more prominent among males and individuals with obesity/overweight. High adherence to the “high vegetable” pattern was also related to lower odds of hypertriglyceridemia and chronic inflammation. No significant association was found between high adherence to "high animal" and "high carbohydrate" patterns and risk of MU. However, high adherence to "high animal" and moderate adherence to "high carbohydrate" nutrient patterns was respectively associated with lower odds of hypertension and IR, respectively. Additional large-scale prospective studies are needed to affirm these results.

### Supplementary Information

Supplementary Table 1.

## Data Availability

The datasets used and analyzed during the current study available from the corresponding author on reasonable request.

## Abbreviations

NP: Nutrient pattern
BDNF: Brain-derived neurotrophic factor
FFQ: Food frequency questionnaire
FBG: Fasting blood glucose
TG: Triglyceride
HDL: High density lipoprotein
LDL: Low density lipoprotein
hs-CRP: High-sensitivity C-reactive protein
MH: Metabolically healthy
MU: Metabolically unhealthy
OR: Odds ratio
CI: Confidence interval
NCDs: Non-communicable diseases
IR: Insulin resistance
MetS: Metabolic syndrome
MUNW: Metabolically unhealthy normal-weight
MUOW: Metabolically unhealthy overweight/obese
MHNW: Metabolically healthy normal-weight
MHOW: Metabolically healthy overweight/obese
BMI: Body mass index
WC: Waist circumference
BP: Blood pressure
IPAQ: International physical activity questionnaire
PA: Physical activity
MUFA: Mono-unsaturated fatty acid
TFA: Trans fatty acid
SES: Socioeconomic status
