# Supplementary material for: Nutrient patterns in relation to metabolic health status and serum levels of brain-derived neurotrophic factor (BDNF) and adropin in adults
Source: Sci Rep. 2024 Feb 26;14:4650. doi: 10.1038/s41598-024-54913-0 (PMC10897437; doi:10.1038/s41598-024-54913-0)
Supplement: Supplementary file 1 — Supplementary Table 1. [file 41598_2024_54913_MOESM1_ESM.docx]

| **Supplementary Table 1****. Factor loadings and explained variances for major nutrient patterns (NPs).^1^** | | | | |
| --- | --- | --- | --- | --- |
|  | Mean intake of participants^2^  (n=527) | Factor loadings | | |
|  |  | NP1 High animal protein | NP2 High vegetable | NP3 High carbohydrate |
| Animal protein (gr) | 46.15 ± 0.83 | 0.92 | - | - |
| Vitamin B_12_ (µg) | 4.12 ± 0.08 | 0.91 | - | - |
| Zinc (mg) | 10.22 ± 0.12 | 0.86 | 0.30 | - |
| SFAs (gr) | 22.31 ± 0.34 | 0.84 | - | - |
| Phosphorus (mg) | 1202.33 ± 14.65 | 0.83 | 0.39 | - |
| Vitamin B_2_ (mg) | 1.98 ± 0.03 | 0.79 | 0.423 | - |
| Cholesterol (mg) | 275.93 ± 5.08 | 0.78 | - | - |
| MUFAs (gr) | 21.76 ± 0.29 | 0.77 | - | 0.31 |
| Calcium (mg) | 924.03 ± 16.19 | 0.73 | 0.37 | - |
| Pantothenic acid (mg) | 5.98 ± 0.07 | 0.71 | 0.54 | 0.25 |
| PUFAs (gr) | 16.04 ± 0.32 | 0.50 | - | 0.32 |
| Sodium (mg) | 3775.35 ± 110.19 | 0.35 | 0.20 | - |
| Vitamin D (µg) | 0.93 ± 0.04 | 0.34 | - | 0.27 |
| Total fiber (gr) | 21.17 ± 0.28 | - | 0.89 | 0.58 |
| Vitamin C (mg) | 198.45 ± 4.39 | - | 0.87 | - |
| Potassium (mg) | 3765.68 ± 45.58 | 0.25 | 0.85 | - |
| TFAs (gr) | 47.70 ± 0.95 | - | 0.77 | - |
| Folate (µg) | 341.86 ± 4.87 | 0.39 | 0.74 | 0.30 |
| Vitamin A (µg) | 1333.73 ± 39.73 | 0.24 | 0.72 | - |
| Magnesium (mg) | 283.86 ± 2.85 | 0.55 | 0.72 | 0.29 |
| Vitamin B_6_ (mg) | 1.80 ± 0.02 | 0.45 | 0.67 | 0.36 |
| Vitamin K (µg) | 151.99 ± 3.98 | 0.23 | 0.66 | - |
| Copper (mg) | 1.49 ± 0.02 | 0.42 | 0.66 | 0.46 |
| Vitamin E (mg) | 6.88 ± 0.14 | 0.31 | 0.51 | 0.33 |
| Manganese (mg) | 3.84 ± 0.08 | - | 0.49 | 0.37 |
| Biotin (µg) | 19.46 ± 0.29 | 0.36 | 0.46 | 0.42 |
| Fluoride (µg) | 21909.21 ± 1049.57 | - | 0.20 | - |
| Vitamin B_1_ (mg) | 2.04 ± 0.02 | 0.21 | 0.36 | 0.84 |
| Plant protein (gr) | 34.65 ± 0.34 | - | 0.41 | 0.77 |
| Selenium (mg) | 0.08 ± 0.002 | - | - | 0.75 |
| Iron (mg) | 17.86 ± 0.18 | 0.20 | 0.32 | 0.74 |
| Niacin (mg) | 22.96 ± 0.20 | 0.45 | 0.26 | 0.73 |
| Chromium (mg) | 0.02 ± 0.00 | - | - | 0.69 |
| Carbohydrate (gr) | 345.73 ± 2.12 | - | 0.55 | 0.67 |
| Sugar (gr) | 106.45 ± 1.95 | - | - | 0.24 |
| Variance explained (%) |  | 25.57 | 23.82 | 15.77 |
| Cumulative explained variance (%) |  | 25.57 | 49.39 | 65.16 |
| ^1^Factor loadings < │0.20│ are not shown for simplicity. The Kaiser–Meyer–Olkin value was 0.88. Factors with Eigen values≥2 were used to extract major NPs.  ^2^ Age, sex, and total energy-adjusted intake of participants are resented as mean ± SE. | | | | |
